# DPANN Archaea and CPR Bacteria: insights into early cellular evolution?

**DOI:** 10.1098/rstb.2024.0096

**Published:** 2025-08-07

**Authors:** Tara A. Mahendrarajah, Anja Spang

**Affiliations:** ^1^ Marine Microbiology and Biogeochemistry, Royal Netherlands Institute for Sea Research, Den Burg, Noord-Holland, The Netherlands; ^2^ Evolutionary & Population Biology (IBED-EPB), University of Amsterdam Faculty of Science, Amsterdam, The Netherlands

**Keywords:** tree of life, archaea, bacteria, last universal common ancestor, CPR, DPANN

## Abstract

Cultivation-independent techniques have facilitated the discovery of a diversity of thus far uncultivated organisms and led to the identification of new branches within the tree of life. This has reshaped the general view of key evolutionary processes and challenged fundamental understandings of cellular evolution. Two diverse and phylogenetically relevant lineages are the Diapherotrites, Parv-, Aenigma-, Nano- and Nanohaloarchaeota (DPANN) archaea and the Candidate Phyla Radiation (CPR) bacteria, originally proposed to form sister groups of all other Archaea and Bacteria, respectively. Members of the DPANN and CPR have reduced genome and cell sizes and incomplete biosynthetic pathways. In addition, the co-cultivation of the first DPANN and CPR representatives with their respective hosts indicates that they comprise many symbionts. While initial work has suggested that these two prokaryotic groups could have important implications for understanding cellular evolution and the complexity of the ancestors of the Archaea and Bacteria, respectively, more recent data have indicated that they may be derived organisms or have evolved in parallel with their partner organisms. Here, we provide insights into debates on the phylogenetic placement of these radiations and discuss implications for cellular evolution and the last universal common ancestor (LUCA) in light of Earth history.

This article is part of the discussion meeting issue ‘Chance and purpose in the evolution of biospheres’.

## 1. Introduction

Cellular life on Earth is extremely diverse. Aside from eukaryotes that comprise much of the visible biosphere, the vast majority of cellular organisms consists of prokaryotes. Prokaryotes are represented by two primary domains of life, the Archaea and Bacteria [1], members of which have colonized most environments on Earth, thriving under a wide range of conditions (e.g. temperature, pH, pressure, nutrient and trace gas composition). In recent years, advances in environmental sampling and (meta-)genome sequencing have provided a window into the uncultivated biosphere, expanding our understanding of microbial diversity and uncovering newly defined branches in the growing tree of life [2–4]. The tree of life is a network that provides insight into evolutionary relationships between and among extant prokaryotes and eukaryotes from a shared ancestor. Contemporary views of the tree of life highlight the significance of prokaryotes, which have shaped planetary ecosystems for most of Earth’s history and underlie the origin of the eukaryotic cell that emerged around 2.0 billion years ago (Ga) [5–11].

**Figure 1.**
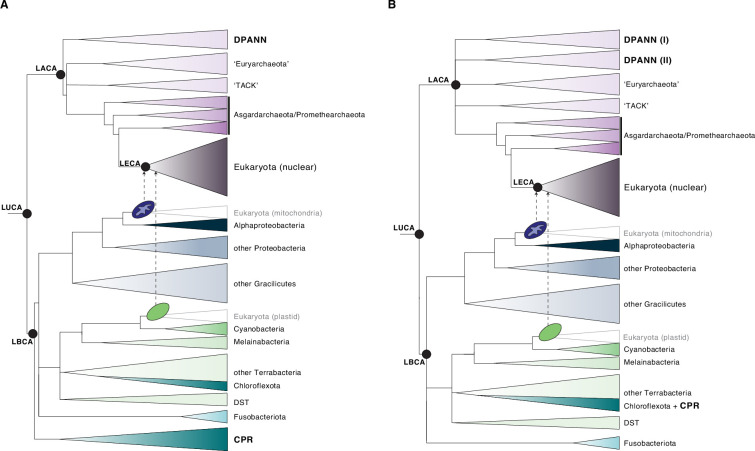
A depiction of the tree of life highlighting shifts in our understanding of the placements of the DPANN archaea and CPR bacteria. The schematic trees are based on recent phylogenetic and molecular dating analyses presented in [19] and [8], and they approximately reflect relative evolutionary distance. (A) Initial phylogenetic inferences positioned the Diapherotrites, Parv-, Aenigma-, Nano-, and Nanohaloarchaeota (DPANN) and the Candidate Phyla Radiation (CPR) clades as the sister clades to all other Archaea and Bacteria, respectively. (B) More recent phylogenetic evidence supports a derived position for the CPR within the Terrabacteria, with the group sharing a common ancestor with the Chloroflexota/Dormibacterota, while the monophyly of DPANN and the root of the archaeal tree remain unresolved. LUCA, LACA, LBCA and LECA refer to the last universal common ancestor, the last archaeal common ancestor, the last bacterial common ancestor and the last eukaryotic common ancestor, respectively. DST refers to the clade comprising Deinococcota, Synergistota and Thermotogota. DPANN (I) refers to DPANN Cluster 1 and DPANN (II) refers to DPANN Cluster 2 [20]. The blue and green oval-shaped cells depict the mitochondrial and plastid endosymbiosis events, respectively.

All known cellular life shares characteristics that indicate descent from a single ancestor or cellular population, more commonly known as the last universal common ancestor (LUCA) [12–14]. The nature of LUCA, its metabolic features and timing of divergence into Archaea and Bacteria [1] are foundational to understanding cellular evolution. The recently discovered DPANN (named after the first discovered lineages: Diapherotrites, Parv-, Aenigma-, Nano- and Nanohaloarchaeota) [15,16] and the CPR (i.e. the Candidate Phyla Radiation, also referred to as Patescibacteria) [16–18] have been suggested to be instrumental for addressing unanswered questions surrounding the earliest periods of cellular evolution. Specifically, both the DPANN and CPR lineages were originally positioned as sister clades to all other Archaea and Bacteria, respectively, in phylogenetic analyses (figure 1A) [3,16,21–23], raising the question as to whether members of these groups may more closely resemble LUCA than other archaeal and bacterial lineages [21,24,25].

However, speculations about a simple ancestor inspired by these findings are problematic for several reasons. First of all, most DPANN and CPR representatives with reduced genomes appear to be symbionts that are obligately dependent on a host organism for growth and survival [17,21,26–31]. It is unclear how a cell with such biosynthetic limitations could have sustained a lifestyle independent from (a) host organism(s) in the primordial environment and give rise to all extant life on Earth. In addition, the genomes of symbionts often evolve at faster rates and are characterized by compositionally biased nucleotide compositions, which can contribute to phylogenetic artefacts such as long branch attraction (LBA) [32,33], where fast-evolving taxa are erroneously attracted towards a distant outgroup unless more complex phylogenetic approaches are employed. Thus, it is possible that the early divergence of DPANN and CPR from all other Archaea and Bacteria, respectively, is a result of such artefacts (figure 1A) [20,34–38]. Even if the early divergence of the ancestors of DPANN and CPR were to be correct, it is important to note that they still share a common ancestor with their respective archaeal and bacterial sister-lineage predominantly [39] comprising organisms with lifestyles not obligately dependent on a host organism (i.e. host-independent) (figure 1A). Therefore, tree topology alone cannot be used to infer whether the last bacterial common ancestor (LBCA) and the last archaeal common ancestor (LACA) had a limited metabolic repertoire and a host-dependent lifestyle or whether they were more reminiscent of a *bona fide* bacterium and archaeon, respectively.

In recent years, the increased quality and quantity of genomic reconstructions have greatly expanded the known diversity of members belonging to both the DPANN and CPR lineages. Their integration into careful phylogenetic and ancestral reconstruction analyses is imperative to gain comprehensive insight into cellular evolution and understand the evolutionary mechanisms that give rise to symbiotic lifestyles. Here, we discuss recent phylogenetic discoveries that challenge the initial view of the early divergence of DPANN and CPR for our understanding of early cellular evolution and the divergence of the major domains of life (Archaea and Bacteria).

## 2. The Candidate Phyla Radiation is a derived sister lineage of the Chloroflexota

The CPR has been suggested to represent a substantial portion of bacterial diversity [3,17,18]. Various phylogenetic reconstructions using different approaches, ranging from traditional concatenation-based methods with a small set of universally conserved ribosomal proteins [3] to supertree methods using a greatly expanded set of marker genes including metabolic genes [23], have initially inferred a placement of this lineage as a sister group to all other bacterial lineages (figure 1A) [3,23]. This finding led to speculations as to whether the small genome-reduced cells, with patchy biosynthetic capabilities, might reflect the cell biology and lifestyle of LBCA and possibly of earlier ancestors along the bacterial stem.

However in recent years, comparative genomic and phylogenetic approaches integrating better-fitting models of evolution and accounting for the various forms of gene transmission including transfers, losses and originations (reviewed in [40]) have challenged the hypothesis of the early divergence of the branch leading to CPR from the rest of the Bacteria, instead resolving them as a sister group to the Chloroflexota within the Terrabacteria (figure 1B) [19,41–43]. For instance, using outgroup-free rooting methods based on gene tree–species tree reconciliations and distinct taxonomic datasets, Coleman *et al.* found a derived position for CPR, with this clade sharing a common ancestor with Chloroflexota and Dormibacterota (figure 1B) [19]. Gene tree–species tree reconciliation approaches [44] furthermore indicated that LBCA was a relatively complex, motile and rod-shaped cell, with a double-membrane and evidence of the presence of several core carbohydrate-processing pathways [19]. This is in agreement with another study that suggested a diderm ancestor of all Bacteria, with a possible loss of the outer membrane along the stem to the CPR/Chloroflexota [43,45]. In turn, current data are best in line with the view that LBCA was a cell similar to extant bacteria, with CPR representing a derived lineage that evolved more recently via genome reduction from a host-independent ancestor.

## 3. Diapherotrites, Parv-, Aenigma-, Nano- and Nanohaloarchaeota, an early diverging lineage with host-independent lifestyles?

Similar to the CPR, the DPANN archaea [16] comprise organisms with ‘ultrasmall’ cells, reduced genomes and limited metabolic capabilities [15,16]. They are proposed to represent a substantial proportion of archaeal diversity [3], currently comprising 10 archaeal phyla [46]. Initial phylogenetic analyses [3,15,16] identified the DPANN as a monophyletic clade that forms a sister group to all other Archaea within the archaeal tree (figure 1A). A more recent outgroup-free rooting analysis also recovered a root between the DPANN and all other Archaea [22]; however, the number of DPANN genomes included in that analysis was limited and does not reflect the currently known diversity of the group. Indeed, a more recent study integrating a better representation of DPANN could not unequivocally determine the position of the root of the archaeal domain, challenging previously proposed scenarios on the group’s phylogenetic position and monophyly (figure 1B) [20,47]. The placement of the root of the archaeal phylogeny remains unresolved, complicating our inferences of LACA (figure 1B). Consequently, the debate over the monophyly of the DPANN, their possible derived nature and co-evolution with their hosts remains.

Lineages such as the Nanohaloarchaeota, which have been identified in hypersaline environments associating with the archaeal class Halobacteria [16,28,48], were initially assigned to the Euryarchaeota [34,48,49]. While the Nanohaloarchaeota regularly cluster within the DPANN and exhibit characteristic traits such as extremely small cell sizes and patchy biosynthetic pathways, critiques have argued that they are erroneously attracted toward the DPANN owing to amino acid composition biases in their proteome, which are a consequence of adaptation to hypersaline environments [34,36]. For example, although Feng *et al.* [36] recovered Nanohaloarchaeota as part of the DPANN based on concatenated marker gene tree analyses, they argued that the clustering of ATP synthase subunits of Nanohaloarchaeota with those of Halobacteria indicates a close phylogenetic relationship of these lineages. In contrast, Wang *et al.* [50] suggested that horizontal transfer of the ATP synthase operon from halobacterial hosts into the nanohaloarchaeal symbionts best explains the relationship of their ATP synthase subunits. In our recent work on the early evolution of the ATP synthase across the tree of life, the catalytic headpiece subunit sequences of the Nanohaloarchaeota cluster with those of other DPANN archaea rather than with Halobacteria, while the non-catalytic headpiece subunit sequences cluster with the Halobacteria [8], likely as a result of compositional attraction or host–symbiont gene transfer. In line with this, a recent study integrating careful modelling of evolutionary processes of halophilic archaeal lineages has confirmed the placement of Nanohaloarchaeota with other DPANN [51] and that certain nanohaloarchaeal and halobacterial protein homologues are phylogenetically attracted to each other due to compositional biases resulting from adaptations to an environment with high salt concentrations [8,51].

It is notable that DPANN lineages seem to form at least two distinct sub-clades (Cluster 1 and Cluster 2), with Cluster 2 lineages currently known to comprise phyla with smaller genomes and more limited metabolic potential [20]. In contrast, the Cluster 1 DPANN comprise, in part, taxa that do not seem to have reduced genomes and are instead predicted to have host-independent lifestyles. For instance, certain members of the Diapherotrites have been proposed to have secondarily evolved greater independence, including the ability to synthesize ATP and various amino acids, nucleotides and cofactors, as a consequence of gene acquisitions from putative bacterial partners [52]. Furthermore, members of the Altiarchaeota do not seem to have reduced genomes; for instance, they are predicted to use the Wood–Ljungdahl pathway (WLP) for carbon fixation [53] and may even serve as hosts for other DPANN, such as members of the Huberarchaeota [54,55]. Altogether, it is therefore possible that the ancestor of the DPANN Cluster 1 lineage may not have been a genome-reduced symbiont. Thus, irrespective of the root placement, current data are consistent with a host-independent LACA. Prospective work integrating a better representation of recently discovered DPANN lineages with outgroup-free rooting methods and ancestral reconstructions will, however, be needed to further validate this hypothesis.

## 4. What have we learned about LUCA and timing of cellular evolution?

Phylogenetic approaches have helped to improve our resolution of the tree of life and the placement of DPANN and CPR, indicating that the ancestors of the Archaea and Bacteria, respectively, were complex organisms similar to extant representatives of these domains [19,22]. This has also allowed us to improve our knowledge of LUCA, the ‘elusive’ last universal common ancestor(s) of all cellular life [56–58]. Piecing together the characteristics of LUCA as well as its timing relative to Earth history is challenging owing to the sparsity of fossils and available biomarkers for prokaryotes. Consequently, inference of LUCA’s metabolism and ecological role relies on phylogenetic and ancestral genome reconstruction approaches that examine gene histories in the context of archaeal and bacterial species phylogenies [14,59–63]. However, owing to the use of varying approaches and datasets to reconstruct the nature of LUCA, these analyses have resulted in distinct perspectives on what LUCA looked like [56]. For instance, some authors have proposed a communal, acellular and/or RNA-based LUCA, while others envisioned LUCA to have been more complex than extant prokaryotes ([14,57,59–61,64–66]; and recently reviewed in [57]). In contrast, some recent studies indicated that LUCA may have been a cellular organism similar to extant prokaryotic life [56,62,63], which would be in agreement with the universality of various cellular features across all organismal life. For example, by analysing genes that are shared by Archaea and Bacteria and broadly distributed amongst extant organisms, Weiss *et al.* proposed gene families that may trace back to LUCA [63]. They inferred LUCA to be a hyperthermophilic chemolithoautotrophic anaerobe that was hydrogen (H_2_)-dependent, could fix carbon dioxide (CO_2_) via the WLP [67,68] and conserve energy via substrate-level phosphorylation coupled to an ATP synthase [63]. Indeed, the WLP is often considered to represent an ancestral carbon fixation pathway as it allows for the generation of ATP during CO_2_ fixation, making it one of the most energetically favourable carbon-fixation pathways [69,70].

However, these findings [63] have been challenged [71–74]. For instance, subsequent work indicated that several of the protein families inferred to have been present in LUCA on the basis of reciprocal domain monophyly may represent false positives, with domain monophyly resulting from undersampling of the protein families [71]. Furthermore, the subsequent phylogenetic inspection of key protein families in Weiss *et al*.’s reconstruction, such as the reverse gyrase and nitrogenase, has challenged the notion of a hyperthermophilic nitrogen-fixing LUCA [71,72,75]. While reciprocal domain monophyly of a protein family was used by Weiss *et al.* as a major criterion for its inference to LUCA [63], the violation of domain monophyly does not exclude the presence of a gene family in LUCA. However, interdomain transfers increase the difficulty of accurately inferring the LUCA proteome.

To overcome this challenge, we recently employed a probabilistic gene tree–species tree reconciliation approach that infers the history of gene duplication, transfer, loss and origination events along a species tree, thereby allowing us to determine the probability of a gene family having been present at any given node in a species tree [40,62]. Our results resolved LUCA’s predicted genome size to approximately 2.75 Mb with 2657 genes, similar in complexity to extant Archaea and Bacteria. The inferred LUCA protein set shares consistencies with the inference of Weiss *et al.* [63], suggesting that LUCA may have been an anaerobic hyperthermophilic organism/cellular population that encoded the WLP and may have been able to grow autotrophically [62]. These results may localize LUCA’s habitat to environments where H_2_ and CO_2_ were readily available [76]. However, it is important to emphasize that the WLP is not unique to autotrophs, with its presence being equally compatible with an organo-heterotrophic or mixotrophic metabolism [62,77,78]. Thus, while the improvement of our understanding of the tree of life and the use of novel computational approaches have recently allowed for more systematic reconstructions of the LUCA proteome, more work is needed to further inform deep cellular evolution and resolve contradictory hypotheses [57].

Linking cellular life’s evolutionary timeline to past geological and biogeochemical data can provide complementary information on the evolution of microbial metabolism. Molecular clock techniques coupled with fossil and biomarker constraints can be used to timescale evolutionary events. However, current molecular clock methods are limited due to sparse prokaryotic fossil evidence necessary for clock calibration and challenges associated with adequately modelling rate variation through time. Alternative approaches are necessary to improve time estimates for prokaryotic evolution. Recently, we addressed some of these challenges [8] by applying an approach referred to as cross-bracing [10]. Genes resulting from ancient duplications and those inherited via endosymbiosis result in node-equivalency, a concept where multiple nodes within a phylogeny (single gene phylogenies or concatenated species phylogenies) correspond to the same evolutionary event. This temporal equivalence provides additional constraints to molecular dating as it allows for bracing corresponding nodes in the phylogeny to the same age. We made use of phylogenetic marker genes encoding protein machinery of which eukaryotes have inherited at least two paralogous forms from their respective archaeal and bacterial ancestors [79,80], such as ribosomal proteins and the ATP synthase. To improve our timing estimates, we combined this novel implementation of the cross-bracing approach with a relative time constraint [81], requiring that the node corresponding to the last plastid common ancestor be younger than the nodes corresponding to the last nuclear and mitochondrial eukaryotic common ancestors [8]. Using this approach, we estimated that LUCA lived between 4.52 and 4.32 Ga. Interestingly, our results also indicated that LBCA lived in an overlapping period from 4.49 to 4.05 Ga, whereas LACA lived from 3.95 to 3.37 Ga. Additional analyses are required to determine whether the estimated age of LACA is the result of poor taxonomic sampling along the archaeal stem or indicates a bottleneck in early archaeal evolution [8]. These findings are consistent with other recent studies using alternative dating methods that resolved the age of LUCA to approximately 4.52−4.48 Ga [5], 4.33−4.09 Ga [62] and a range of ages between 4.40−4.22 Ga [82] (table 1). Others have obtained younger ages for LUCA, e.g. a range of ages from 4.48 to 3.93 Ga [84] and 4.04−3.98 Ga [83] (table 1). LUCA’s age is highly sensitive to, and clashes against, the maximum age calibration applied to the root node (table 1) (see also [5,42]). Furthermore, results may differ depending on whether the late heavy bombardment (LHB) (*ca*. 3.9 Ga) [85], the earliest evidence of liquid water (*ca* 4.4 Ga) [86,87], the Moon-forming impact (4.52−4.51 Ga) [88] or the earliest evidence of life based on biomarkers and microfossils (4.1−3.5 Ga) [89–91] is applied as soft and/or hard age constraints [5,8,42,62,82–84] (table 1). It seems that increasing evidence questions the occurrence of a cataclysmic LHB in favour of a more drawn-out period of bombardments [85,92], which may have allowed life to emerge any time after the moon-forming impact and survive periods of bombardment in locally shielded environments. Further improvements in molecular dating approaches combined with new planetary, fossil and biomarker data will allow us to resolve more closely the time when LUCA thrived.

**Table 1.** An overview of estimated age(s) and age ranges of LUCA (last universal common ancestor) and applied root calibrations that were reported in some recent studies mentioned in this opinion article.

study	inferred age of LUCA (Ga)	root node calibration (Ma)	description	references
Betts *et al.*, 2018	4.52–4.48	4520−3347	Any life on Earth would have emerged after sterilization post Moon-forming impact (max); age of the Strelley Pool formation (min).	[5]
Parsons *et al.*, 2021	4.04–3.98	3800 (conservative) 4100 (liberal)	Both root node calibrations are based on the earliest evidence of life on Earth; the conservative calibration represents the consensus age, whereas the liberal calibration represents the earliest age reported in the literature. Root ages inferred using the liberal calibration exceeded 4.5 Ga and were not considered in the analysis.	[83]
Mateos *et al.*, 2023	4.48 4.05 3.93	3800 (conservative) 4100 (liberal)	Both root node calibrations are based on the earliest evidence of life on Earth; the conservative calibration represents the consensus age, whereas the liberal calibration represents the earliest age reported in the literature. Different age inferences using the conservative calibration (reported here) correspond to different molecular clock models. Root ages inferred using the liberal calibration were between 5.3 and 6.0 Ga, exceeding the age of Earth.	[84]
Mahendrarajah *et al.*, 2023	4.52-4.32	4520−3347	Any life on Earth would have emerged after sterilization post Moon-forming impact (max); age of the Strelley Pool formation (min).	[8]
Boden *et al.*, 2024	4.40-4.22 4.40-4.34 4.40-4.29	4400−3500	Earliest evidence of liquid water on Earth (max); earliest evidence of life based on microfossils (min). Different age inferences (reported here) correspond to different molecular clock models.	[82]
Moody *et al.*, 2024	4.33-4.09	4520−3347	Any life on Earth would have emerged after sterilization post Moon-forming impact (max); age of the Strelley Pool formation (min).	[62]

## 5. Conclusions

Resolving the phylogenetic position of DPANN archaea and CPR bacteria is crucial for our understanding of cellular evolution. Improved phylogenetic methods have revised the placement of the CPR bacteria as a derived sister-group of Chloroflexota, indicating genome streamlining from an ancestor that was not obligately host-dependent [19,37,41,43]. In contrast, while several different analyses have resolved the DPANN to be a sister group of all other Archaea [3,15,16,22,23], their monophyly and their exact root placement within the Archaea remain debated. Resolving the archaeal tree of life will thus be crucial to further improve our understanding of early cellular and archaeal evolution. For example, improved gene tree–species tree aware methods combined with a better sampling of extant archaeal biodiversity [40] would allow the rooting of an expanded archaeal phylogeny in the absence of an outgroup and predict the protein content of the archaeal ancestor. Gene family histories can furthermore help to elucidate the evolution of genome-streamlining (and potentially expansion) patterns across the tree of life. Integrating data from DPANN genomes into evolutionary reconstructions of key metabolic pathways such as the WLP [77], methanogenesis and anaerobic methane/alkane oxidation [93–96] will be key to further link biogeochemistry with cellular evolution. We expect progress in our understanding of early evolution through increased focus on multidisciplinary endeavours that brings together experts from a variety of fields including not only biogeochemistry, geology and chemistry but also synthetic biology and data sciences. For instance, the integration of novel approaches rooted in machine learning, such as structural predictions and phylogenetics, may help overcome some of the limitations of sequence-based reconstructions [40,97]. Finally, resurrecting and experimentally studying ancestral proteins will allow us to better understand their function under simulated early Earth conditions and test predictions [98–100].

## Data Availability

This article has no additional data.
